# Mr. Broughton, on Hydrophobia

**Published:** 1808-06

**Authors:** S. D. Broughton

**Affiliations:** Pupil to Mr. G. Freer, Surgeon. New Street, Birmingham


*To the Editors of the Medical and Physical Journal.*

GENTLEMEN,

THE frequency of a disease, which has generally baffled all medical skill, induces me to publish a Case of Hydrophobia that terminated fatally, and another wherein that dreadful disease was averted.

Though I have not yet passed the ordeal of my profession, yet I trust the importance of the Cases (communicated to me by my brother, who resides in the East Indies) will be a sufficient apology for my requesting a place for them in your valuable Journal.

I am, &c.

S. D. BROUGHTON,

Pupil to Mr. G. FREER, Surgeon.

*New Street, Birmingham, May* 6, 1808.

---

ON

ON the 27th of January, 1807, the Soobadar, or native commissioned officer of the escort, was roused from his tent at day-break by the screams of a child; and upon going out, he saw his own dog, a large half mastiff, tearing a little boy, about seven years of age. In endeavouring to rescue the child, he himself was bitten in the right hand, in three different places; two of the wounds were very deep.

Some time previous to this accident, the camp had been very much annoyed by the dogs of a neighbouring Rajah's army flocking over to them, and biting several people, some of whom died. This was a farther inducement to the persons who were assembled at this accident to destroy the dog, which they accordingly did immediately.—The dog was a favourite among the officers, and appeared perfectly well the evening before the accident.

The Soobadar and the boy were taken directly to the surgeon of the escort, Mr. Macauley. The child was so dreadfully mangled about the belly and loins, as to prevent the possibility of applying any remedies against Hydrophobia, so was delivered to the native surgeon, with orders to clean his wounds, &c. Mr. Macauley proceeded to administer such remedies as he thought might tend to save the Soobadar's life. His own experience did not furnish him with any mode of treatment which might prevent or cure Hydrophobia; he had read of large doses of mercury being given with success; this plan he resolved to adopt, in hopes of averting the dreadful complaint, which he expected would otherwise seize his patient.

A tournequet being fixed upon the arm, the wounds were deeply scarified, and filled with eau de luce (the spiritus salis ammoniacæ succinatus of the new Nomenclature); the wounds were then well washed with warm water, poured from an height, and refilled with eau de luce. This plan was continued for half an hour, during which the flow of blood was considerable. A pot of water, as hot as could be borne, into which two hands full of common salt had been thrown, was procured, and the Soobadar's hand plunged into it. The temperature of the water was kept up by fresh supplies. In this the hand lay immersed for nearly five hours, and the quantity of blood lost was considerable; two small arteries were divided by the scarificators. The tournequet was now removed, and the wounds well filled with an epispastic ointment.

At eight o'clock he took thirty grains of calomel; the same quantity was repeated at eleven o'clock; again at one one, and at five o'clock. In the morning he took some doses of eau de luce; and in the course of the day four doses of musk, each dose containing twelve grains, were administered. At sun-set he felt himself perfectly easy in every respect. He said, he did not think the dog was mad; but, even if he were, that he felt himself quite confident in the remedies applied.

On the morning of the 28th, he felt himself well; the hand had not been painful, and he had had one stool.— Matter was formed in the wounds, which were cleansed, and filled with fresh ointment. At the hours of seven and eleven, A. M. and three and seven, P. M. during this day, he took fifteen grains of calomel each time. In the evening he felt his mouth to be rather sore.

29th. The wounds discharged freely. During this day he took three doses of calomel, each containing ten grs. In the evening his mouth became very sore and was much swelled.

Feb. 13. The patient's mouth quite well. The wounds continued to discharge freely till the 20th, when the applications were removed, and the Soobador returned to his duty perfectly well.

In the mean time, the child had been dressed in a common manner; its wounds were quite healed, and it had played about with other children of the camp, apparently in very good health, till the 11th of March, when there was too much reason to believe that the child was going to suffer Hydrophobia. Restlessness and a dislike to water were at this time the principal indications.

March 12th. As soon as water was presented to him he fell into violent convulsions.

On the 14th Mr. Macauley visited him, and found him feeble and languid, his pulse low and tremulous, and his skin yellow. He was greatly irritated at being asked to drink. When persuaded to try to drink, he instantly became convulsed. After the lapse of a few minutes he would recover, and appear perfectly sensible. At three, P. M. the boy expired with every symptom of Hydrophobia.

No one now entertained a doubt of the dog's madness. The fears of all were excited for the life of the respectable old Soobadar, who had been upwards of thirty years in the service. He himself felt no alarm whatever; and day after day, and week after week, passed on without any derangement of his health. On the 27th of July, 1807,

1807, he was still perfectly well, and quite confident in the plan his surgeon had adopted.

Should the hopes of his friends prove fallacious, and the Soobadar be attacked with symptoms of Hydrophobia, it is intended that opium, musk, and the cold bath, shall form the chief means of cure. The opium is proposed to be introduced into the system principally by friction.

